# A Comparison of Players’ and Coaches’ Perceptions of the Coach-Created Motivational Climate within Youth Soccer Teams

**DOI:** 10.3389/fpsyg.2017.00109

**Published:** 2017-02-01

**Authors:** Nina Elise Møllerløkken, Håvard Lorås, Arve Vorland Pedersen

**Affiliations:** Department of Neuromedicine and Movement Science, Faculty of Medicine and Health Sciences, NTNU – Norwegian University of Science and TechnologyTrondheim, Norway

**Keywords:** motivation, attrition, adolescents, sport, football, dropout, boys, girls

## Abstract

The coach-created motivational climate within youth sports teams has been shown to be of great importance for the quality of youths’ sports experiences as well as their motivation for continuing or discontinuing sport participation. While the player’s perspective on motivational climates has been studied extensively, the coach’s perspective has received considerably less attention. Thus, little is known about the concordance of perceptions of the motivational climate between coaches and their players, or the lack thereof. The purpose of the present study was to directly compare players’ and coaches’ perceptions of the motivational climate within their respective teams. To this end, 256 male and female soccer players (15–17 years of age) from 17 different teams and their coaches (*n* = 29) responded to the Perceived Motivational Climate in Sports Questionnaire-2 (PMCSQ-2). The study design included responses from both coaches and players to the same questionnaire, and both groups were aware of the other part’s participation. Statistical analyses revealed significant differences between players’ and coaches’ perceptions of the motivational climate. Specifically, players of both sexes perceived the motivational climate to be significantly more performance-oriented and significantly less mastery-oriented compared with the coaches. These findings may advance our understanding of the coach-athlete relationship, and may be of importance for understanding players’ motivation for persistence or discontinuation of the sport.

## Introduction

Organized sports are a prominent achievement context for youths, and large numbers of children and youths participate in organized sports every year (Stuntz and Weiss, 2009). Participation in a sport provides youths with the opportunity to engage in physical activity, which in turn results in a number of positive health effects, well-documented through extensive research (Warburton et al., 2006; Khan et al., 2012; Eime et al., 2015). In addition, participation in sports has beneficial effects upon a variety of motor, mental, and social factors (Donaldson and Ronan, 2006; Janssen and LeBlanc, 2010; Eime et al., 2013). Furthermore, a high level of physical activity in youth increases the likelihood of being physically active in adulthood (Telama et al., 2005; Huotari et al., 2011).

Additional effects can be obtained by participating in team sports, as compared with individual sports. For example, Schumacher Dimech and Seiler (2011) found a reduction in social anxiety over time in children participating in team sport compared with those participating in an individual sport or no sport, and Boone and Leadbeater (2006) found team sport involvement to be positively associated with social acceptance, while it partially mediated risks of depressive symptoms. In their comprehensive review, Eime et al. (2013) concluded that team sport engagement seemed to be associated with improved health effects compared to engagement in individual sports.

The coach is arguably a very important person influencing youths’ general sport experience. The coach’s organization, facilitation, and behavior in practice sessions and competition has been shown to influence athletes’ motivation to participate (Fry and Gano-Overway, 2010; Rottensteiner et al., 2013; Smith et al., 2016a). Coaches may positively affect individuals’ abilities, beliefs, and enjoyment, and induce a desire for challenging and mastery experiences (Mageau and Vallerand, 2003; Weiss et al., 2009). It has even been suggested that the coach may enhance youths’ personal development and life skills (Gould et al., 2007). On the other hand, coaches have the potential to induce anxiety and burnout in athletes, and ultimately dropout from the sport (Smith et al., 2007; Weiss et al., 2009).

The coaches’ influence has been attributed, in part, to the motivational climate they create through the transfer of attitudes and values, as well as their recognition and evaluations, and is linked to the athletes’ learning and performance (Joesaar et al., 2012). Therefore, coaches play a critical role in either hindering or strengthening an athlete’s involvement in, and motivation for, sports (Treasure and Roberts, 1995; Alvarez et al., 2009).

A commonly applied theoretical perspective when studying motivational climates in youth sport is the achievement goal theory (AGT) (Nicholls, 1984). AGT attempts to explain how an individual cognitively processes and develops his or her views about achievement under various social contexts and influences. The term motivational climate refers to the structure of the learning environment in achievement settings that affects an individual’s participation, thoughts, feelings, and behavior, and reflects the actions of coaches and parents, such as their use of rewards, punishments, and feedback (Ames and Archer, 1988; Joesaar et al., 2012). A mastery-oriented climate is characterized by valuing the learning process, such as cooperating with others, and trying new solutions, whereas a performance-oriented climate values outperforming others, and is characterized using external rewards, and by discriminating in favor of the best athletes (Duda and Nicholls, 1992).

Indeed, studies have shown that a coach-cultivated motivational climate centering on task goals (i.e., a mastery-oriented climate), fosters more positive cognitive and emotional responses and adaptive achievement patterns among athletes than does an ego-involving environment (i.e., a performance-oriented climate) (Balaguer et al., 1999; Mageau and Vallerand, 2003). Athletes’ perception of a mastery-oriented motivational climate in sports settings is associated with a variety of positive factors concerning thoughts and attitudes toward the activity and increased intrinsic motivation and social values related to the activity, which are of importance to persistence and continuation of a sport (Ntoumanis and Biddle, 1999; Ommundsen et al., 2005). More specifically, the perception of a mastery-oriented climate is associated with sports persistence, whereas the perception of a performance-oriented climate is associated with dropout (Boiche and Sarrazin, 2009; Iwasaki and Fry, 2013). For example, Fry and Gano-Overway (2010) found a positive relationship between youth soccer players’ perception of a caring, or mastery-oriented, climate and their intentions of engaging in soccer in the future, while Cumming et al. (2007) found that the motivational climate was, in fact, more important than the win-loss percentage of the team for young athletes’ enjoyment and desire to continue playing for the coach.

The coach may have every intention of creating a mastery-oriented climate, but may not be able to do so. This could be due to a lack of pedagogical or educational skills, which are not always possessed by coaches just because they have extensive sporting experience, or coaching badges (Werthner and Trudel, 2006). It is well known that coaches are more frequently chosen because of their sport-specific competence than their interpersonal skills (see Gilbert et al., 2009). Furthermore, as shown by Stebbings et al. (2011, 2012), coaches’ personal well- or ill-being affects their coaching behavior. Further, a coach may have the intention of creating a mastery-oriented motivational climate, and be capable of doing so in less competitive practice sessions, but may not be able to transfer this climate to a match situation, in which the pressure is greater (Smith et al., 2016a).

Despite extensive study of motivational climates, the topic of how coaches perceive motivational climates, and whether the coach’s perception of the motivational climate within the team (created by himself or herself) is in concordance with the perceptions of the players has been under-researched. Divergences in perceptions of the motivational climate could potentially be a reason for dissatisfaction among players, and ultimately, why players choose to drop out from the team, and potentially, from the sport altogether.

Among the relatively few studies that have focused on the coach’s perspective, Stebbings et al. (2011, 2012) studied the effect of coaches’ psychological well- and ill being on their coaching behavior across a number of sports and found that coaches’ well-being positively predicted their autonomy-supportive behavior toward their athletes, while ill-being was associated with a controlling coaching style. Stebbings et al.’s studies, however, included coaches only. In previous studies, when comparing players and coaches’ perceptions of motivational climates, only correlations have been reported. Such studies have included findings of a moderate positive relationship between players’ and coaches’ perceptions of the motivational climates (Boyce et al., 2009), or little such relationship at all (Curtis et al., 1979). Neither the magnitude nor the direction of differences in perceptions of motivational climates or coach behavior was elaborated upon in those studies. Correlations between players’ and coaches’ perceptions of both climates (task-involving and ego-involving) in the research of Boyce et al. were only approximately 0.50, and Curtis et al. found generally low correlations on perceptions of coach behavior between coaches and their young baseball players. The results of both studies thus indicate that the convergence of perceptions may be less than complete.

Smith et al. (2016b) argued that including coaches’ perceptions of the motivational climate in research studies would provide a more comprehensive assessment of the environment. The results of their study, which included both players’ and coaches’ perceptions, compared with reports of independent observers, showed that there were moderate positive associations between players’ and coaches’ perceptions of the maladaptive and controlling behaviors of the coaches, but less agreement with respect to the more positive, empowering dimensions of the motivational climate.

One possible (albeit speculative) reason for the overall lack of empirical studies on the coach’s perspective on motivational climates may be that researchers fear that answers may be biased, as coaches are aware of the motivational climate desired by their players and how their behavior may negatively influence their players (see also above, Ntoumanis, 2012; Smith et al., 2016a). This awareness might prime coaches into underestimating the performance-orientation of their approach and instead portray their team as being typified by the more socially desirable mastery-oriented climate, an effect known as social desirability bias, which is reported in self-report measures across all social sciences (see Fisher, 1993). Stebbings et al. (2011, 2012) attempted to control for such social desirability bias, in that they presented sport coaches in their study with a social desirability scale, assessing their tendency to respond in a socially desirable manner. Another potential strategy for securing more honest answers from coaches is that of Smith et al. (2016b), in which coaches and players responded to the same questionnaire, assessing the coaching environment within their teams.

The present study applied a similar design to that of Smith et al. (2016b), having coaches and players answer the same questionnaire, the Perceived Motivational Climate in Sports Questionnaire-2 (PMCSQ-2) (Newton and Duda, 1993), and each group was aware of this fact. Thus, the purpose of the present study was to extend the previous literature on perceptions of the motivational climate in youth soccer, by examining both players’ and coaches’ perceptions of the motivational climate within their teams and comparing them. Unlike previous studies that reported correlations of perceptions (Curtis et al., 1979; Boyce et al., 2009), the present study directly compared each player’s responses to those of his or her own coach and tested for differences across groups.

Girls have been shown to be more in favor of a task-oriented motivational climate, compared with boys (Kavussanu and Roberts, 2001; Lemyre et al., 2001), and it has been argued that they may also be somewhat differently motivated compared with boys (see Donaldson and Ronan, 2006). Furthermore, girls are more prone to dropping out from soccer (Møllerløkken et al., 2015). Therefore, analyses were separated by gender in order to investigate possible differences in girls’ versus boys’ convergence of perceptions of the motivational climate when compared with their coaches. In addition, demographic background variables were included in the questionnaire in order to investigate the possible effects of team or squad structure, as well as coaches’ soccer-specific and formal education, and their coaching experience. Coaches’ backgrounds may be important for both their perceptions of, and their knowledge about motivational climates, as well as their capacity to create a certain climate (Mageau and Vallerand, 2003; Boiche and Sarrazin, 2009; Rottensteiner et al., 2013).

## Materials and Methods

### Participants

Players and coaches belonging to 17 different soccer teams were recruited from two regions in Norway through a mailed request. Inclusion criteria for both players and coaches were that they had to have completed at least one season with the current team in order for the motivational climate to be well established. As the literature does not give any formal guidelines on how long it takes for the motivational climate to be established (Reinboth and Duda, 2006), this was done as a precaution. Across the teams in the sample, 47.1% were male players with an average of 26 players (range 12–41) per team. Seventy percent of the squads were divided based upon skill level, and the coaches reported an average of eight players (range 0–31) dropping out in the past 3 years.

#### Coaches

The sample consisted of 29 coaches (one female) with a mean (range) age of 40.9 (21–57) years. They had been coaching the current team for a mean (range) of 4.8 (1–10) years, and 62% had also coached other teams before. The overall mean (range) of coaching experience was 8.9 (1–30) years. The overall education level was completed high school (52%) or college education. For coaching-specific education, 55% had a national level license, and 14% had a Union of European Football Association Level 3 (UEFA-B) coaching license. Thirty-one percent of the coaches received a salary from their club.

#### Female Players

One hundred twenty-eight girls with a mean (range) age of 15.7 (15–17) years participated in the study. They had been with their current team for a mean (range) of 5.6 (1–11) years and had been playing soccer for a mean (range) 7.3 (1–11) years. Forty-four percent reported that they were playing on their first team.

#### Male Players

One hundred twenty-eight boys with a mean (range) age of 15.6 (15–17) completed the questionnaire. They had been with their current team for a mean (range) of 5.9 (1–12) years, and their total soccer experience was a mean (range) 9.1 (3–15) years. Forty-six percent reported that they were playing on their first team.

### Procedure

For each team, all questionnaires were distributed within a single practice session, in which completion took approximately 20 min. All data were collected in the pre-season period between January and March.

### Questionnaires

The first part of the questionnaire contained demographical questions concerning the respondents’ age, gender, number of years playing soccer, and number of years on the current team, as well as whether the squad was divided into several teams (1st, 2nd, or 3rd), and whether this selection was based on players’ performance levels. The coaches also gave information about their general educational level and their coach-specific education or training.

### The Perceived Motivational Climate in Sports Questionnaire-2 (PMCSQ-2)

Perception of the motivational climate within the team was assessed by a Norwegian version of the PMCSQ-2. The PMCSQ-2 has been used in several studies related to the motivational climate in sports, and initial studies using the PMCSQ-2 have found it to have adequate internal reliability and factorial validity for youths as well (Newton and Duda, 1993; Newton et al., 2000).

The PMCSQ-2 is a 33-item inventory, and consists of two higher-order factors measuring *performance*- (16 items) and *mastery*- (17 items) oriented motivational climates. Each of these two higher-order factors consists of three lower-order factors (the result being six lower-order factors). For the higher-order factor of mastery-oriented motivational climate, the lower-order factors are labeled *cooperative learning*, *effort*, *and improvement*, and *each player has an important role*. For the performance-oriented motivational climate, the lower-order factors are labeled *intra-team member rivalry*, *unequal recognition from the coach*, and *intolerance of mistakes*. The stem for each item is; “On this team…,” and responses were indicated on a 5-point Likert scale anchored by strongly disagree (1) and strongly agree (5), giving data on an ordinal level.

### Translation Procedure

The PMCSQ-2 was translated into Norwegian and adapted to soccer. The translation was based on the original English version, as well as a version that was slightly modified for use on dancers (Carr et al., 2003) that had already been translated into Norwegian. Two graduate sociologists reviewed the translated version of the questionnaire along with the original version, and in this way, back-translation was secured, which is of importance for the construct validity (Chapman and Carter, 1979). The aim of the present study, however, was to compare the players’ perceptions of the motivational climate to that of their coaches. Therefore, it was necessary to reformulate the phrases in the questionnaire in order for them to refer to the coaches. The questions in the player’s version of the questionnaire (the original one) were thus inverted so that they reflected the coach’s perspective, much in the same way as done in the work of Stebbings et al. (2011) and Smith et al. (2016b). An illustration of this is the phrase, “On this team, the coach wants us to try new skills,” which, after reformulating, would say, “On my team, I encourage the players to try new skills.” The same was done for all items of the PMCSQ-2.

### Statistical Analyses

Kolmogorov–Smirnov tests, histograms, and Q-Q plots were applied to confirm normality assumptions of the distributions in the eight factor scores from the PMCSQ-2. Despite evidence of the factorial validity of the PMCSQ-2 (see e.g., Newton et al., 2000; Olympiou et al., 2008), the Norwegian version of the inventory was subjected to confirmatory factor analysis (CFA) in order to confirm the expected hierarchical, second order factor structure. This analysis was conducted on the players’ data, as there were too few coaches in the sample to complete the CFA on this sub-sample. In this model, the mastery-oriented climate serves as the correlated higher-order factor of the subscales cooperative learning, effort and improvement, and each player has an important role. Furthermore, the higher-order factor performance-oriented climate captures shared variance between the lower-order factors intra-team member rivalry, unequal recognition, and intolerance of mistakes. The multi-dimensional hierarchical model was evaluated against various types of overall goodness-of-fit indices for the constructed model: chi-squared (χ^2^) (Barrett, 2007), root mean square error of approximation (RMSEA) (MacCallum et al., 1996), and normed fit index (NFI) (Bentler and Bonett, 1980). The CFA were conducted with the IBM AMOS 23.0.0 software (IBM SPSS, US).

The players’ scores on the higher and lower-order factors obtained from the PMCSQ-2 were analyzed against demographical variables with a general linear model (GLM) MANOVA. For this full factorial model, the variables gender and whether they played on the 1st, 2nd, or 3rd team were designated as fixed factors, and variables of age, years of playing soccer, and years of playing on the team as covariates. Similarly, the coaches’ perceptions of the motivational climate were subjected to a similar GLM MANOVA procedure with demographical variables of receiving a salary from the club (yes or no), general educational level, and coach-specific education as fixed factors, and variables of age, years of coaching the current team, and overall years of coaching experience as covariates. In all pairwise multiple comparisons, the alpha was Bonferroni corrected, and the partial eta squared ($\eta_{p}^{2}$) was applied as measure of effect size.

As a first step, in order to examine the association between coach and athletes’ perceptions of the motivational climate, bivariate correlations were used to examine the relationships between player- and coach-perceived higher- and lower-order environment dimensions and are reported as Pearson’s product-moment correlation coefficients (*r*). Further statistical analyses incorporated the fact that the nature of the data, with players nested within their teams and respective coaches, requires multilevel analysis in order to examine the convergence between players’ and coaches’ perceptions of the motivational climate within their teams. The specified linear mixed model with the restricted maximum likelihood method included 29 coaches at level 2 and 256 athletes at level 1.

The first step in the multilevel modeling involved running baseline component models to determine the amount of variance attributed to the grouping of athletes within teams for each of the two higher-order factors, and for each of the six lower-order factors. Intra-class correlation values (ICC) of 8.8 and 22.2% for mastery- and performance-oriented climate, respectively, 1.8% for cooperative learning, 11.1% for effort and improvement, 10.4% for each player has an important role, 19.2% for intra-team member rivalry, 13.8% for unequal recognition, and 27.3% for intolerance of mistakes, suggested that a significant amount of variance in the athletes’ reports of the eight environment dimensions could be attributed to the grouping of athletes within teams (i.e., within coach). Thus, further examination of the convergence between the players’ and coaches’ responses were examined by specifying these as fixed effects in the model and conducting Bonferroni corrected pairwise comparisons with Cohen’s *d* as the measure of effect size. MANOVAs and multilevel modeling were conducted using Predictive Analytics Software (PASW, IBM, US; previously SPSS) Version 23.0.0 with *p* < 0.05 as the statistical significance criterion.

## Results

Descriptive statistics (mean, SD) for the players’ and coaches’ responses on the higher and lower-order factors from the PMCSQ-2 can be found in **Table 1**.

**Table 1:** Descriptive statistics [Mean (SD)] for players and coaches and internal consistency for higher- and lower-order factors from the Perceived Motivational Climate in Sports Questionnaire-2 (PMCSQ-2).

		Players		
		
	α^1^	Girls (*n* = 128)	Boys (*n* = 128)	Coaches (*n* = 29)
*Higher-order factors*				
Mastery-oriented climate	0.88	72.3 (7.5)	70.5 (9.8)	79.1 (7.4)
Performance-oriented climate	0.88	36.3 (10.3)	41.8 (11.5)	29.7 (9.1)
*Lower-order factors*				
Cooperative Learning	0.75	12.3 (2.0)	12.05 (2.4)	13.8 (1.0)
Effort and Development	0.76	35.2 (3.5)	34.3 (4.3)	37.6 (2.1)
All have an Important Role	0.79	24.7 (3.6)	24.1 (4.3)	27.7 (2.4)
Intra-Team Member Rivalry	0.61	10.8 (3.1)	11.8 (3.2)	8.3 (3.9)
Uneven Recognition (coach)	0.86	14.5 (5.7)	17.5 (6.4)	11.4 (3.9)
Intolerance of mistakes	0.74	10.9 (3.5)	12.5 (4.2)	9.9 (3.6)


*^1^Cronbach’s coefficient alpha.*

### Factor Structure of the PMCSQ-2

The CFA of the expected hierarchical, second order factor structure resulted in acceptable goodness-of-fit indices (χ^2^ = 11.3, *df* = 5, *p* < 0.05; RMSEA = 0.07, 90% CI Low/High = 0.009, 0.119; NFI = 0.98) (Bentler and Bonett, 1980; MacCallum et al., 1996; Barrett, 2007). The covariance between the higher-order factors of mastery- and performance-involving climates was -0.59, and the loadings of the subscales onto the higher-order factor mastery-oriented climate ranged from 0.83 to 0.85, whereas the loadings of the designated subscales for the performance-oriented climate factor ranged from 0.74 to 0.86. All factor loadings were statistically significant. Cronbach’s coefficient alpha for internal consistency was 0.88 for the higher-order factors and in the range of 0.61–0.86 for the lower-order factors. Although the lower end of the alpha values was somewhat below the suggested 0.70 criteria (Cronbach, 1951) for internal consistency (0.61 for the factor *Intra-team member rivalry*, comprising three of the items from the PMCSQ-2), all factors fitted the CFA model and the pattern of results regarding player/coach divergence was similar for each lower-order factor (outlined below).

### Players’ Responses on the PMCSQ-2

The GLM for the two higher-order factors indicated a significant difference (*F* = 4.32, *df* = 1, *p* < 0.05, $\eta_{p}^{2}$ = 0.023) in boys’ and girls’ perceptions of the performance-oriented climate dimension, in which boys reported a mean score of 6.6 (95% CI: 0.35–12.97) higher compared to the girls. No such difference was obtained for the mastery-oriented climate factor (*F* = 0.82, *df* = 1, *p* > 0.05, $\eta_{p}^{2}$ = 0.01). For the lower-order factors, boys reported significantly higher scores compared to girls on the subscale unequal recognition (*F* = 5.65, *df* = 1, *p* < 0.05, $\eta_{p}^{2}$ = 0.03), with a mean difference of 4.2 (95% CI: 0.71–7.65), and a significant mean difference of 2.3 (95% CI: 0.03–4.57) on the subscale intolerance of mistakes (*F* = 3.90, *df* = 1, *p* < 0.05, $\eta_{p}^{2}$ = 0.02). There were no significant differences in girls’ vs. boys’ scores on the other subscales. Furthermore, there were no significant differences in players scores in relation to whether they played on the 1st, 2nd, or 3rd team, or related to how many years they had been playing soccer. Significant effects of players age (15, 16, or 17 years old) was found (in which the younger players reported lower scores) on the higher-order factor performance-oriented climate (*F* = 5.36, *df* = 1, *p* < 0.05, $\eta_{p}^{2}$ = 0.03) and the lower-order factors effort and improvement (*F* = 6.82, *df* = 1, *p* < 0.05, $\eta_{p}^{2}$ = 0.04) as well as unequal recognition (*F* = 4.75, *df* = 1, *p* < 0.05, $\eta_{p}^{2}$ = 0.03). Lastly, the number of years they had been playing on their current team was significantly associated with the higher-order dimension mastery-oriented climate factor (*F* = 4.63, *df* = 1, *p* < 0.05, $\eta_{p}^{2}$ = 0.02) and the lower-order factor effort and improvement (*F* = 5.49, *df* = 1, *p* < 0.05, $\eta_{p}^{2}$ = 0.03).

### Coaches’ Responses on the PMCSQ-2

Analysis of the coaches’ perceptions of the motivational climate with GLM MANOVA indicated that there were no significant differences (*p* > 0.05) in either higher- or lower-order factors between coaches with different general education levels (secondary school up to more than 4 years in university) or between coaches with or without soccer-specific coaching education (grassroot or UEFA-licenses). Furthermore, pairwise comparisons demonstrated that none of the variables *receiving a salary or not*, *age*, *years of coaching the current team*, and *years of overall coaching experience* induced significant differences (*p* > 0.05) on any of the eight motivational climate factor scores.

### Coaches’ vs. Players’ Responses on the PMCSQ-2

Pearson’s *r* for the association between coaches’ and players’ perceptions of the motivational climate, depicted in **Table 2**, indicated moderate significant correlation coefficients for the higher-order factor mastery-oriented climate and the lower-order factors effort and development and intra-team member rivalry.

**Table 2:** Inter-correlations between players’ and coaches’ scores on higher- and lower-order factors from the PMCSQ-2.

	Pearson product-moment correlations (*r*)
*Higher-order factors*	
Mastery-oriented climate	**0.39**^∗^
Performance-oriented climate	0.28
*Lower-order factors*	
Cooperative Learning	0.34
Effort and Development	**0.40**^∗^
All have an Important Role	0.14
Intra-Team Member Rivalry	**0.46**^∗∗^
Uneven Recognition (coach)	0.15
Intolerance of mistakes	0.25


*Significant correlations (^∗^*p* < 0.05, ^∗∗^*p* < 0.01) in bold.*

Further analysis, in which players’ and coaches’ responses were specified as fixed effects in nested multilevel models, indicated a significant overall effect on the responses on PMCSQ-2 that was dependent upon whether a player or coach answered the questionnaire. For the higher-order factor mastery-oriented climate, the players scored statistically significantly lower, on average 7.5 (95% CI for difference = 4.4–10.9), compared to the coaches (*t* = 4.3, *df* = 283, *p* < 0.001, *d* = 0.55) and significantly higher on the higher-order factor performance-oriented climate (*t* = 4.6, *df* = 283, *p* < 0.001, *d* = 0.51), on average 9.4 (95% CI for difference = 5.1–13.7).

Multilevel analysis of the six lower-order factors indicated a significantly lower score (mean difference 1.5, 95% CI = 0.7–2.3) for the players compared to the coaches on the mastery-oriented climate subscales cooperative learning (*t* = 3.7, *df* = 283, *p* < 0.001, *d* = 0.44), effort and development (mean difference 2.8, 95% CI = 1.4–4.3, *t* = 3.8, *df* = 283, *p* < 0.001, *d* = 0.45) and all have an important role (mean difference 3.3, 95% CI = 1.8–4.6, *t* = 4.4, *df* = 283, *p* < 0.001, *d* = 0.52). Furthermore, statistically significant higher scores amongst the players compared to the coaches were observed on the performance-oriented climate subscales intra-team member rivalry (mean difference 3.0, 95% CI = 1.8–4.2, *t* = 4.9, *df* = 283, *p* < 0.001, *d* = 0.58), uneven recognition (mean difference 4.6, 95% CI = 2.3–6.9, *t* = 3.9, *df* = 283, *p* < 0.001, *d* = 0.46), and intolerance of mistakes (mean difference 1.9, 95% CI = 0.4–3.4, *t* = 2.5, *df* = 283, *p* < 0.05, *d* = 0.30).

## Discussion

The purpose of the present study was to directly compare players’ and coaches’ perceptions of the motivational climate within their youth soccer teams. Furthermore, possible effects of demographic variables upon these relationships were investigated within both groups. The study applied a Norwegian version of the commonly applied PMCSQ-2 questionnaire for assessing the motivational climate, in which CFA of the players’ data suggested the expected two-level factorial structure. The results showed that there was a divergence between coaches’ and players’ perceptions in that coaches perceived the motivational climates as significantly higher in mastery-orientation, and significantly lower in performance-orientation, compared with their players. Aside from a gender difference, in which boys reported higher scores on factors associated with a performance-oriented climate, other demographic variables from players (age, division of the squad, which team the player belongs to) or coaches (formal and sport-specific education) did not affect the overall pattern of results.

Even though previous studies with similar designs have found correlations between players’ and coaches’ perceptions of the motivational climate to be rather low (Curtis et al., 1979; Boyce et al., 2009; Smith et al., 2016b), such divergences of perceptions on direct comparison, as shown in the present study, have not been previously reported.

The results may be accurate, in that there is a divergence of perceptions between players and coaches about the motivational climate within the team, or they might signal biased reporting from coaches, players, or both. If the results are accurate, coaches may wrongly perceive that they create a mastery-oriented climate while creating a climate that is more performance-oriented, which the players correctly perceive. On the other hand, coaches may create a mastery-oriented climate, as they report, but this is wrongly perceived as more performance-oriented by the players. Such misperceptions may be due to unintended behaviors by coaches because of lack of pedagogical or educational skills or experience in otherwise soccer-competent individuals (Werthner and Trudel, 2006; Gilbert et al., 2009), or it may be due to coaches’ psychological ill-being, which has been shown by Stebbings et al. (2011, 2012) to predict controlling behaviors toward their players. The present results thus support the assumption set out by Pensgaard and Roberts (2002), that the coach may have the best intentions of focusing on a mastery-oriented climate, but still the athlete perceives the climate to be performance-oriented perhaps due to unintended behaviors of the coach. If correct, our results indicate that this assumption also for youth soccer players of both genders.

Coaches may also, seek to create a motivational climate for the team that is in accordance with their personal ambitions and views about coaching, and less in accordance with players’ wishes or official goals set by clubs or associations, as was suggested by Jones and Wallace (2005). In such cases, it is possible that coaches’ responses are biased, and that they are underreporting the performance-orientation within their team. Coaches may seek to portray the motivational climate as being more mastery-oriented, as they know that this would be more desirable and socially acceptable. On the other hand, it is also possible that players are biased and portray the performance orientation of the motivational climate more extremely, perhaps as a signal of their disagreement and/or dissatisfaction with the motivational climates, and/or the coach’s (or coaches’) behaviors. From the present results, it is not possible to reach a conclusion about such possible biases. However, evidence supports that if there were any bias or misperceptions regarding the actual climate, it would be among the coaches, as Smith et al. (2015) found scores on an objective measure of the motivational climate (the Multidimensional Motivational Climate Observation System, MMCOS) to be more consistent with athletes’ reports than with those of their coaches. Whatever the cause, however, such biased reporting would seem to support the fact that coaches create a more performance-oriented motivational climate than what is desired by the players, and since it is the players who are motivated, or not, by the climate, their perceptions would be the ones that matter the most. Thus, if coaches wish to succeed with their teams, they should try to create a motivational climate that is more in accordance with the players’ preferences and wishes. Players who are dissatisfied with the motivational climate of the team, or with coaching behaviors associated with exaggerated performance orientation, may choose to leave the team, and even the sport altogether (Fry and Gano-Overway, 2010).

The boys who participated in this study tended toward perceiving a higher performance-orientation than the girls. This is in line with previous research that has repeatedly found female athletes to perceive the motivational climate created by the coach as being more task-involving or mastery-oriented compared with their male counterparts (Kavussanu and Roberts, 1996; White et al., 1998; Carr and Weigand, 2001; Vazou et al., 2006), and probably also reflects an actual difference in motivational climates within girls’ and boys’ teams. In a similar vein, studies have reported higher ego-orientation and lower task-orientation in male players than in female players across a variety of team sports (Kavussanu and Roberts, 2001; Lemyre et al., 2001). These gender differences, associated with players’ perceptions of the motivational climate, can be explained by a general trend of a more competitive environment that emphasizes winning and outperforming others in male teams, which might expose players to performance-oriented practices (Musch and Grondin, 2001). Another postulated explanation is that the sports arenas provide boys with an opportunity to show their masculinity and gain popularity by peers, by demonstrating their strength and athletic skill (Klomsten et al., 2004). A third possibility concerns gender differences in competence-judging criteria, as boys tend to evaluate their competence in line with the characteristics of a performance-oriented climate, whereas girls are known to use more self-referenced criteria, e.g., skill improvement, in line with the characteristics of a mastery-oriented climate (Horn et al., 1993). Clearly, establishing the specific nature of causes and effects of these gender differences in team sports, especially in terms of dropout, is an important area for further research.

The overall perceptions of the motivational climate drawn from the present sample of coaches did not systematically vary as a function of completed formal soccer coaching training or general education level. Although this finding might indicate that the coaching style is unaffected by these variables, it must be considered that a relatively low proportion of coaches were categorized as *without formal coaching training* or of *lower general educational level*. Previous research has shown that interventions aimed at getting coaches to facilitate a mastery-oriented climate in sports are effective without being very costly or time consuming. These interventions are associated with changes in athletes’ anxiety and perceptions of the coaches being more mastery-oriented (Smith et al., 2007). Furthermore, dropout rates have been found to be higher for non-trained coaches compared to coaches with formal sport-specific education (Rottensteiner et al., 2013). As we did not include any specific information about the content of the coaches’ formal training, we can only speculate whether the motivational climate was part of the curriculum.

Vazou (2010) demonstrated that a coach’s gender may have an effect upon players’ perceptions of the motivational climate. Vazou’s results indicated that athletes perceived female coaches to promote a more task-involving, that is, mastery-oriented climate, and less of an ego-involving or performance-oriented climate compared to male coaches. Unfortunately, because only one female coach participated in the present study, we were unable to examine this potential impact of gender. An open question for further study, therefore, is whether there also are gender differences in perceptions of motivational climates among coaches, and whether female coaches’ perceptions of the motivational climate within their teams are more in accordance with those of their players.

### Implications

A divergence of players’ and coaches’ perceptions of the motivational climate within their youth soccer team may indicate that motivational climates within teams are more performance-oriented and less mastery-oriented than the coaches report. As players’ motivations are dependent on the motivational climate that the coaches create, their perceptions would seem to matter more, and thus, the motivational climates should be adjusted so that they are more in accordance with players’ preferences. Players, and especially girls, tend to favor more mastery-oriented climates, thus the climates within the teams included in the present study may in fact be a cause of dissatisfaction among players, which may help in explaining why so many players, especially girls, drop out from the sport. From the present findings, it can be argued that coaching education should emphasize the importance of the motivational climate within a team, and should encourage the creation of a more mastery-oriented motivational climate within their teams.

### Limitations and Future Research Directions

The present study did not include any objective measure that would have indicated which of the groups’ (players or coaches) perceptions of the motivational climates were correct, and did not control for social desirability bias. However, as argued above, any divergence would signal some issues within the team, and therefore would bias reporting from any group. The present study applied the PMCSQ-2 with slight modifications (inverting the questions to fit the coaches’ perspective), similar to previous studies (Curtis et al., 1979; Stebbings et al., 2011, 2012; Smith et al., 2016b). However, in the present study, the direction and magnitude of the divergence was tested. Thus, the present design could motivate further studies targeting the generalizability of the current findings. In particular, a possible line of research would be to examine the perceived motivational climate reported by players no longer playing or those considering dropping out, and compare their perceptions with those of their current or past coaches. In the current study, the CFA was only applied to the players’ data due to a limited *n* in the sub-sample of coaches. Thus, evaluating the psychometrical properties of the Norwegian version of the PMCSQ-2 awaits further study. Substantial and potentially significant differences in the factor structure of players and coach’s data could also shed further light on the divergence in perception of the motivational climate.

## Conclusion

In the present study, coaches and their players perceived the motivational climate within their teams differently in that the coaches reported a more mastery-oriented and less performance-oriented motivational climate than did their players. It is suggested that coaches, inadvertently or on purpose, may create a climate that is more in accordance with their personal preferences, and not necessarily in accordance with the preferences and wishes of their players. Players, and especially girls, tend to favor more mastery-oriented climates. Thus, the climates within the teams included in the present study may in fact be a cause of dissatisfaction among players, and may help in explaining why so many players, especially girls, drop out from the sport. Coaching education should therefore emphasize the importance of the motivational climate within a team, and should encourage coaches to create more mastery-oriented motivational climates within their teams.

## Ethics Statement

The study was approved by The Norwegian National Research Ethics Committee. Information letters about the study, along with a request for respondents, were mailed to each team’s contact person. When agreeing to participate in the study, the teams were contacted again and given further information about the study in the guise of a written information sheet. Completed questionnaires were put in closed envelopes that were each given a code to ensure the respondents’ anonymity. The participants were above the age of 15 which, in accordance with The Norwegian National Research Ethics Committee, does not introduce a requirement for parental consent.

## Author Contributions

Conception and design of study: NM, AP, and HL; acquisition of data: NM; analysis and/or interpretation of data: NM, AP, and HL; drafting the manuscript: NM, AP, and HL; revising the manuscript critically for important intellectual content: NM, AP, and HL; approval of the version of the manuscript to be published: NM, AP, and HL.

## Conflict of Interest Statement

The authors declare that the research was conducted in the absence of any commercial or financial relationships that could be construed as a potential conflict of interest.

## References

[B1] Alvarez M. S. Balaguer I. Castillo I. Duda J. L. (2009). Coach autonomy support and quality of sport engagement in young soccer players. *Span. J. Psychol.* 12 138–148. 10.1017/S1138741600001554 19476227

[B2] Ames C. Archer J. (1988). Achievement goals in the classroom: students’ learning strategies and motivation processes. *J. Educ. Psychol.* 80 260–267. 10.1037/0022-0663.80.3.260

[B3] Balaguer I. Duda J. L. Crespo M. (1999). Motivational climate and goal orientations as predictors of improvement, satisfaction and coach ratings among tennis players. *Scand. J. Med. Sci. Sports* 9 381–388. 10.1111/j.1600-0838.1999.tb00260.x 10606104

[B4] Barrett P. (2007). Structural equation modelling: adjudging model fit. *Pers. Individ. Dif.* 42 815–824. 10.1371/journal.pone.0147257

[B5] Bentler P. M. Bonett D. G. (1980). Significance tests and goodness-of-fit in the analysis of covariance structures. *Psychological Bulletin* 88 588–600. 10.1037/0033-2909.88.3.588

[B6] Boiche J. C. S. Sarrazin P. G. (2009). Proximal and distal factors associated with dropout versus maintained participation in organized sport. *J. Sport Sci. Med.* 8 9–16. PMC3737782 24150550

[B7] Boone E. M. Leadbeater B. J. (2006). Game on: diminishing risks for depressive symptoms in early adolescence through positive involvement in team sports. *J. Res. Adolesc.* 16 79–90. 10.1111/j.1532-7795.2006.00122.x

[B8] Boyce B. A. Gano-Overway L. A. Campbell A. L. (2009). Perceived motivational climate’s influence on goal orientations, perceived competence, and practice strategies across the athletic season. *J. Appl. Sport Psychol.* 21 381–394.

[B9] Carr S. Phil M. Wyon M. (2003). The impact of motivational climate on dance students’ achievement goals, trait anxiety, and perfectionism. *J. Dance Med. Sci.* 7 105–114.

[B10] Carr S. Weigand D. A. (2001). Parental, peer, teacher and sporting hero influence on the goal orientations of children in physical education. *Eur. Phys. Educ. Rev.* 7 305–328. 10.1177/1356336X010073005

[B11] Chapman D. W. Carter J. F. (1979). Translation procedures for the cross cultural use of measurement instruments. *Educ. Eval. Policy Anal.* 1 71–76. 10.3102/01623737001003071

[B12] Cronbach L. J. (1951). Coefficient alpha and the internal structure of tests. *Psychometrika* 16 297–334. 10.1007/bf02310555

[B13] Cumming S. P. Smoll F. L. Smith R. E. Grossbard J. R. (2007). Is winning everything? The relative contributions of motivational climate and won-lost percentage in youth sports. *J. Appl. Sport Psychol.* 19 322–336. 10.1080/10413200701342640

[B14] Curtis B. Smith R. E. Smoll F. L. (1979). Scrutinizing the skipper: a study of leadership behaviors in the dugout. *J. Appl. Psychol.* 64 391–400. 10.1037/0021-9010.64.4.391

[B15] Donaldson S. J. Ronan K. R. (2006). The effects of sports participation on young adolescents’ emotional well-being. *Adolescence* 41 369–389. 16981623

[B16] Duda J. L. Nicholls J. G. (1992). Dimensions of achievement motivation in schoolwork and sport. *J. Educ. Psychol.* 84 290–299. 10.1037/0022-0663.84.3.290

[B17] Eime R. M. Harvey J. T. Charity M. J. Casey M. M. van Uffelen J. G. Z. Payne W. R. (2015). The contribution of sport participation to overall health enhancing physical activity levels in Australia: a population-based study. *BMC Public Health* 15:806 10.1186/s12889-015-2156-9 PMC4545912 26290046

[B18] Eime R. M. Young J. A. Harvey J. T. Charity M. J. Payne W. R. (2013). A systematic review of the psychological and social benefits of participation in sport for children and adolescents: informing development of a conceptual model of health through sport. *Int. J. Behav. Nutr. Phys. Act.* 10:98 10.1186/1479-5868-10-98 PMC3751802 23945179

[B19] Fisher R. J. (1993). Social desirability bias and the validity of indirect questioning. *J. Consum. Res.* 20 303–315. 10.1086/209351

[B20] Fry M. D. Gano-Overway L. A. (2010). Exploring the contribution of the caring climate to the youth sport experience. *J. Appl. Sport Psychol.* 22 294–304. 10.1080/10413201003776352

[B21] Gilbert W. Lichtenwaldt L. Gilbert J. Zelezny L. Côté J. (2009). Developmental profiles of successful high school coaches. *Int. J. Sports Sci. Coach.* 4 415–431. 10.1260/174795409789623928

[B22] Gould D. Collins K. Lauer L. Chung Y. (2007). Coaching life skills through football: a study of award winning high school coaches. *J. Appl. Sport Psychol.* 19 16–37. 10.1080/10413200601113786

[B23] Horn T. S. Glenn S. D. Wentzell A. B. (1993). Sources of information underlying personal ability judgement in high school athletes. *Pediatr. Exerc. Sci.* 5 263–274.

[B24] Huotari P. Nupponen H. Mikkelsson L. Laakso L. Kujala U. (2011). Adolescents physical fitness and activity as predictors of adulthood activity. *J. Sports Sci.* 29 1135–1142. 10.1080/02640414.2011.585166 21777154

[B25] Iwasaki S. Fry M. D. (2013). The efforts of sport psychology professionals to assist sport administrators in evaluating youth sport programs. *Sport Psychol.* 27 360–371. 10.1123/tsp.27.4.360

[B26] Janssen I. LeBlanc A. G. (2010). Systematic review of the health benefits of physical activity and fitness in school-aged children and youth. *Int. J. Behav. Nutr. Phys. Act.* 7 1–16. 10.1186/1479-5868-7-40 20459784 PMC2885312

[B27] Joesaar H. Hein V. Hagger M. S. (2012). Youth athletes’ perception of autonomy support from the coach, peer motivational climate and intrinsic motivation in sport setting: one-year effects. *Psychol. Sport Exerc.* 13 257–262. 10.1016/j.psychsport.2011.12.001

[B28] Jones R. L. Wallace M. (2005). Another bad day at the training ground: coping with ambiguity in the coaching context. *Sport Educ. Soc.* 10 119–134. 10.1080/1357332052000308792

[B29] Kavussanu M. Roberts G. C. (1996). Motivation in physical activity contexts: the relationship of perceived motivational climate to intrinsic motivation and self-efficacy. *J. Sport Exerc. Psychol.* 18 264–280. 10.1123/jsep.18.3.264

[B30] Kavussanu M. Roberts G. C. (2001). Moral functioning in sport: an achievement goal perspective. *J. Sport Exerc. Psychol.* 23 37–54. 10.1123/jsep.23.1.37

[B31] Khan K. M. Thompson A. M. Blair S. N. Sallis J. F. Powell K. E. Bull F. C. (2012). Sport and exercise as contributors to the health of nations. *Lancet* 380 59–64. 10.1016/S0140-6736(12)60865-4 22770457

[B32] Klomsten A. T. Skaalvik E. M. Espnes G. A. (2004). Physical self-concept and sports: do gender differences still exist? *Sex Roles* 50 119–127. 10.1023/B:SERS.0000011077.10040.9a

[B33] Lemyre P. N. Roberts G. C. Ommundsen Y. Miller B. W. (2001). “Sportspersonship in soccer: the role of achievement goals and gender,” in *Proceedings of the International Society of Sport Psychology (ISSP) Xth World Congress of Sport Psychology* Vol. 5 Skiathos, 149–151.

[B34] MacCallum R. C. Browne M. W. Sugawara H. M. (1996). Power analysis and determination of sample size for covariance structure modeling. *Psychol. Methods* 1 130–149. 10.1037/1082-989X.1.2.130

[B35] Mageau G. A. Vallerand R. J. (2003). The coach-athlete relationship: a motivational model. *J. Sports Sci.* 21 883–904. 10.1080/0264041031000140374 14626368

[B36] Møllerløkken N. E. Lorås H. Pedersen A. V. (2015). A systematic review and meta-analysis of dropout rates in youth soccer. *Percept. Mot. Skill* 121 1–11. 10.2466/10.PMS.121c23x0 26595205

[B37] Musch J. Grondin S. (2001). Unequal competition as an impediment to personal development: a review of the relative age effect in sport. *Dev. Rev.* 21 147–167. 10.1006/drev.2000.0516

[B38] Newton M. Duda J. L. (1993). The perceived motivational climate in sport questionnaire-2: construct and predictive validity. *J. Sport Exerc. Psychol.* 15 172–172. 10.1123/jsep.15.2.172

[B39] Newton M. Duda J. L. Yin Z. (2000). Examination of the psychometric properties of the perceived motivational climate in sport questionnaire - 2 in a sample of female athletes. *J. Sports Sci.* 18 275–290. 10.1080/026404100365018 10824644

[B40] Nicholls J. G. (1984). Achievement motivation: conceptions of ability, subjective experience, task choice, and performance. *Psychol. Rev.* 91 328–346. 10.1037/0033-295X.91.3.328

[B41] Ntoumanis N. (2012). “A self-determination theory perspective on motivation in sport and physical education: current trends and possible future research directions,” in *Advances in Motivation in Sport and Exercise* Vol. 3 eds Roberts G. C. Treasure D. C. (Champaign, IL: Human Kinetics), 91–128.

[B42] Ntoumanis N. Biddle J. (1999). A review of motivational climate in physical activity. *J. Sports Sci.* 17 643–665. 10.1080/026404199365678 10487465

[B43] Olympiou A. Jowett S. Duda J. L. (2008). The psychological interface between the coach-created motivational climate and the coach-athlete relationship in team sports. *Sport Psychol.* 22 423–438. 10.1123/tsp.22.4.423

[B44] Ommundsen Y. Roberts G. C. Lemyre P. N. Miller B. W. (2005). Peer relationships in adolescent competitive soccer: associations to perceived motivational climate, achievement goals and perfectionism. *J. Sports Sci.* 23 977–989. 10.1080/02640410500127975 16195049

[B45] Pensgaard A. M. Roberts G. C. (2002). Elite athletes’ experiences of the motivational climate: the coach matters. *Scand. J. Med. Sci. Sports* 12 54–59. 10.1034/j.1600-0838.2002.120110 11985767

[B46] Reinboth M. Duda J. L. (2006). Perceived motivational climate, need satisfaction and indices of well-being in team sports: a longitudinal perspective. *Psychol. Sport Exerc.* 7 269–286. 10.1016/j.psychsport.2005.06.002

[B47] Rottensteiner C. Laakso L. Pihlaja T. Konttinen N. (2013). Personal reasons for withdrawal from team sports and the influence of significant others among youth athletes. *Int. J. Sports Sci. Coach.* 8 19–32. 10.1260/1747-9541.8.1.19

[B48] Schumacher Dimech A. Seiler R. (2011). Extra-curricular sport participation: a potential buffer against social anxiety symptoms in primary school children. *Psychol. Sport Exerc.* 12 347–354. 10.1016/j.psychsport.2011.03.007

[B49] Smith N. Quested E. Appleton P. R. Duda J. L. (2016a). Observing the coach-created motivational environment across training and competition in youth sport. *J. Sports Sci.* 35 149–158. 10.1080/02640414.2016.1159714 27055568

[B50] Smith N. Tessier D. Tzioumakis Y. Fabra P. Quested E. Appleton P. R. (2016b). The relationship between observed and perceived assessments of the coach- created motivational environment and links to athlete motivation. *Psychol. Sport Exerc.* 23 51–63. 10.1016/j.psychsport.2015.11.001

[B51] Smith N. Tessier D. Tzioumakis Y. Quested E. Appleton P. Sarrazin P. (2015). Development and validation of the multidimensional motivational climate observation system. *J. Sport Exerc. Psychol.* 37 4–22. 10.1123/jsep.2014-0059 25730888

[B52] Smith R. E. Smoll F. L. Cumming S. P. (2007). Effects of a motivational climate intervention for coaches on young athletes’ sport performance anxiety. *J. Sport Exerc. Psychol.* 29 39–59. 10.1123/jsep.29.1.39 17556775

[B53] Stebbings J. Taylor I. M. Spray C. M. (2011). Antecedents of perceived coach autonomy supportive and controlling behaviors: coach psychological need satisfaction and well-being. *J. Sport Exerc. Psychol.* 33 255–272. 10.1123/jsep.33.2.255 21558583

[B54] Stebbings J. Taylor I. M. Spray C. M. Ntoumanis N. (2012). Antecedents of perceived coach interpersonal behaviors: the coaching environment and coach psychological well- and ill- being. *J. Sports Exerc. Psychol.* 34 481–502. 10.1123/jsep.34.4.481 22889690

[B55] Stuntz C. P. Weiss M. R. (2009). Achievement goal orientations and motivational outcomes in youth sport: the role of social orientations. *Psychol. Sport Exerc.* 10 255–262. 10.1016/j.psychsport.2008.09.001

[B56] Telama R. Yang X. Viikari J. Valimaki I. Wanne O. Raitakari O. (2005). Physical activity from childhood to adulthood - A 21 year tracking study. *Am. J. Prev. Med.* 28 267–273. 10.1016/j.amepre.2004.12.003 15766614

[B57] Treasure D. C. Roberts G. C. (1995). Applications of achievement goal theory to physical education: implications for enhancing motivation. *Quest* 47 475–489. 10.1080/00336297.1995.10484170

[B58] Vazou S. (2010). Variations in the perceptions of peer and coach motivational climate. *Res. Q. Exerc. Sport* 81 199–211. 10.1080/02701367.2010.10599667 20527305

[B59] Vazou S. Ntoumanis N. Duda J. L. (2006). Predicting young athletes’ motivational indices as a function of their perceptions of the coach- and peer-created climate. *Psychol. Sport Exerc.* 7 215–233. 10.1016/j.psychsport.2005.08.007

[B60] Warburton D. E. R. Nicol C. W. Bredin S. S. D. (2006). Health benefits of physical activity: the evidence. *Can. Med. Assoc. J.* 174 801–809. 10.1503/cmaj.051351 16534088 PMC1402378

[B61] Weiss M. R. Amorose A. J. Wilko A. M. (2009). Coaching behaviors, motivational climate, and psychosocial outcomes among female adolescent athletes. *Pediatr. Exrec. Sci.* 21 475–492. 10.1123/pes.21.4.475 20128366

[B62] Werthner P. Trudel P. (2006). A new theoretical perspective for understanding how coaches learn to coach. *Sport Psychol.* 20 198–212. 10.1123/tsp.20.2.198

[B63] White S. A. Kavussanu M. Guest S. M. (1998). Goal orientations and perceptions of the motivational climate created by significant others. *Phys. Educ. Sport Pedagogy* 3 212–228. 10.1080/1740898980030209

